# Autophagy Induced by the N-Terminus of the Classic Swine Fever Virus Nonstructural Protein 5A Protein Promotes Viral Replication

**DOI:** 10.3389/fmicb.2021.733385

**Published:** 2021-08-25

**Authors:** Chengcheng Zhang, Xiuling Wang, Jiahao Sun, Mengjiao Guo, Xiaorong Zhang, Yantao Wu

**Affiliations:** College of Veterinary Medicine, Jiangsu Co-innovation Center for the Prevention and Control of Important Animal Infectious Disease and Zoonoses, Yangzhou University, Yangzhou, China

**Keywords:** classical swine fever virus, nonstructural protein 5A, autophagy, protein interaction, light chain 3

## Abstract

Although classic swine fever virus (CSFV) infection has been reported to induce autophagy, the specific induced mechanism remains unrevealed. Nonstructural protein 5A (NS5A) of CSFV is a multiphosphorylated protein with multiple functions to regulate viral replication and the host cell immune responses. Herein, we demonstrated that CSFV NS5A could induce cellular autophagy and promote viral replication. In the current study, we showed that NS5A expression significantly increased the levels of autophagy-related genes (ATGs), including light chain 3 (LC3), ATG5, and Beclin 1; conversely, degradation of P62/sequestosome 1 (SQSTM1) was observed by Western blotting. The number of autophagy-like vesicles was also obviously increased in NS5A-expressing cells, as analyzed by transmission electron microscopy (TEM). Furthermore, we observed the co-localization of the NS5A and LC3 proteins by confocal immunofluorescence analysis. Direct binding of NS5A to the autophagy-related LC3 protein was confirmed by coimmunoprecipitation *in vivo* and by a GST pulldown assay *in vitro*. Through segmentation and point mutation research on the NS5A protein, we found that the N-terminal region and the phosphorylation of amino acids 81 and 92 of the NS5A protein were essential for inducing autophagy. Finally, we demonstrated that the LC3 protein had a positive effect on CSFV replication. These findings emphasize a previously unascertained interaction relationship between NS5A and LC3 in the autophagy process. Furthermore, our research revealed a new role of CSFV NS5A, particularly its N-terminal amino acids serine 81 and serine 92, as a critical regulator of CSFV-induced autophagy and have significance for extending our understanding of the CSFV-autophagy interplay.

## Introduction

Classic swine fever is an acute, feverish, and highly contagious infectious disease caused by classic swine fever virus (CSFV), which is also one of the World Organization for Animal Health notifiable diseases that has caused serious problems for the swine industry (Moennig et al., 2003; Abid et al., 2019). Infection with the CSFV Shimen strain is usually characterized by high morbidity and mortality, while infection with the CSFV C-strain causes chronic disease with atypical symptoms (Meyers and Thiel, 1996; Tautz et al., 2015).

Classic swine fever virus is a positive single-stranded RNA virus belonging to the *Pestivirus* genus within the *Flaviviridae* family (Casciari et al., 2020). The virus genome encodes a single polyprotein that is processed into four structural (C, E^rns^, E1, and E2) and eight nonstructural proteins [N^pro^, P7, NS2, NS3, NS4A, NS4B, nonstructural protein 5A (NS5A), and NS5B] by host and viral proteases (Lamp et al., 2011). In recent years, a host of studies have been carried out on the pathogenic mechanism of CSFV; for instance, CSFV infection can induce autophagy (Yoo et al., 2018), but which CSFV proteins play a role in the process of autophagy is unclear.

Classic swine fever virus NS5A is a multifunctional phosphorylated protein including 497 amino acids that participate in viral propagation and regulates cellular signaling pathways. NS5A can be divided into three domains, domains I (aa 1–268), II (aa 269–497), and III (aa 29–268), which are segregated by low complexity sequence 1 and the membrane localization sequence, respectively (Zhang et al., 2015). NS5A is a multiphosphorylated protein whose five key phosphorylated amino acids are serine 15 (15S), serine 81 (81S), serine 92 (92S), threonine 274 (274T), and threonine 401 (401T), as determined by software analysis. Studies have shown that CSFV NS5A can regulate viral RNA replication by binding to NS5B and the 3'UTR and can also interact with cellular heat shock proteins 70 and 27 (HSP70 and HSP27) to negatively regulate the replication of CSFV and inhibit the secretion of inflammatory cytokines induced by activation of the NF-kB signaling pathway (Chen et al., 2012; Sheng et al., 2014; Zhang et al., 2015; Xu et al., 2020). The CSFV NS5A protein also effects on the cellular unfolded protein response to accelerate viral propagation and CSFV internal ribosome entry site-dependent translation (Xiao et al., 2009; Chengcheng et al., 2020).

Autophagy is conserved cellular machinery for consuming autophagic proteins or organelles, recycling them into vesicles, which fuse with lysosomes to form autophagosomes and degrade their encapsulated contents to maintain cell homeostasis (Klionsky and Emr, 2000; Ravanan et al., 2017). As a cell death process, autophagy plays a crucial part in regulating diseases (Wu et al., 2015). Several dozen genes involved in autophagy, such as multiple autophagy-related genes (ATGs), including ATG5, ATG7, microtubule-related protein light chain 3 (LC3), P62/sequestosome 1 (SQSTM1), and Beclin 1, are often used as markers of autophagy in experiments (Hirano and Kanno, 2020). Furthermore, these genes usually mediated the formation of autophagosomes and combine with the lysosomes (Klionsky, 2007; Lin et al., 2010).

Recent studies have shown that the HCV NS5A protein, another *Flaviviridae* family member, can inhibit the apoptosis of cells by increasing the expression of the ATG Beclin 1 and can also degrade hepatocyte nuclear factor 1α in lysosomes through chaperone-mediated autophagy (Matsui et al., 2018; Quan et al., 2019). Additionally, there are some reports show that the CSFV NS5A protein is closely related to autophagy (Pei et al., 2014); however, its mechanism of inducing autophagy is largely unclear.

This study investigated the mechanism by which CSFV NS5A is involved in cell autophagy and the effect of LC3 expression on CSFV replication. We found that the N-terminus and the key amino acids serine 81 and serine 92 of the CSFV NS5A protein play a critical role in inducing cellular autophagy. These findings highlight a previously unascertained role of the CSFV NS5A protein in regulating autophagy and the interaction relationship between NS5A and LC, which may mediate the process of inducing cellular autophagy to promote virus infection and the quantity of viral production, which enlarged our understanding of the multifunction’s NS5A protein and the mechanism of virus utilize autophagy to facilitate viral replication.

## Materials and Methods

### Chemicals and Plasmids

Fetal bovine serum (FBS, Gibco-Invitrogen, 10270-106), Dulbecco’s modified Eagle’s medium (DMEM), and T4 DNA ligase were purchased from Thermo Fisher Scientific. Restriction endonucleases, including *Bam*HI, *Eco*RI, and *Xho*I, were purchased from New England Biolabs. SDS-polyacrylamide gel electrophoresis (SDS-PAGE) loading buffer (5×) was obtained from Beyotime. The NS5A gene was cloned into the pDsGFP-N1 vector, and the LC3 gene was cloned into the pDsRed-Monomer-N1 vector using the *Bam*HI and *Eco*RI restriction endonucleases. The CSFV NS5A protein was divided into three segments and cloned into the pcDNA3.1(+) vector with a Flag tag using *Bam*HI and *Xho*I restriction enzymes. LC3 was cloned into the pcDNA3.1(+) vector with a Myc tag using *Bam*HI and *Xho*I restriction enzymes.

### Virus and Cell Culture

The virus used in the current study is the CSFV Shimen strain. The porcine kidney cell line PK-15 and the human renal epithelial cell line 293T were stored in our laboratory and cultured in DMEM supplemented with 10% FBS, 10kU/ml penicillin 1% 10mg/ml streptomycin, and 250μg/ml amphotericin B at 37°C in a 5% CO_2_ incubator (Zhang et al., 2015).

### Antibodies

The primary antibodies were as follows: anti-LC3B (Abcam, ab-229327), anti-ATG5 (Bioss, bs-4005R), anti-Beclin 1 (Bioss, bs-1353R), and anti-GAPDH (Bioss, bs-0755R). Rabbit anti-DDDDK tag, anti-P62/SQSTM1, anti-MYC-tag, and mouse anti-GST tag antibodies were obtained from Proteintech. The secondary antibodies were goat anti-rabbit IgG/HRP (Bioss, bs-0259G-HRP) or goat anti-mouse IgG (H+L)/HRP (Bioss, bs-40296G-HRP).

### Western Blotting

Protein was acquired from PK-15 cells using radioimmunoprecipitation assay (RIPA) lysis buffer. Phenylmethanesulfonyl fluoride (PMSF) was added within minutes before use to a final concentration of 1mM, followed by incubation on ice until the cells were lysed completely. The lysates were centrifuged at 12,000rpm for 5min, and the supernatant was transferred into a new tube. The protein was stored at −20°C. Before the experiment, the protein samples were added to an appropriate amount of 5× SDS-PAGE loading buffer then boiled at 100°C for 5min. Proteins were separated by SDS-PAGE and transferred onto PVDF membranes by a semidry transfer instrument. After blocking with TBST containing 5% nonfat milk powder at 37°C for 1.5h or at 4°C 12h, the membranes were incubated with specific antibodies against GAPDH, LC3, ATG5, BECN 1, or P62 overnight at 4°C and then with secondary antibodies (diluted at 1:10,000) for 3h at 37°C. The protein bands were detected by using an ECL kit. Protein images were obtained with Bio-Rad software.

### Quantitative Real-Time PCR

After the NS5A protein was expressed in PK-15 cells, total RNA was extracted from cells using an RNA kit and reverse transcribed to cDNA. Changes in ATGs were observed and normalized to a housekeeping gene, GAPDH. Quantitative real-time PCR was performed using SYBR Green Master Mix. The reaction procedure was as follows: 98°C for 5min, followed by 40cycles of 98°C for 5s, 58°C for 35s, and 72°C for 40s.

### Transmission Electron Microscopy

Cells were grown in six-well plates; the cell culture medium was removed after 48h, and the cells were washed with cold PBS. A cell scraper was used to scrape the cells gently in one direction, which were transferred into 1.5ml Eppendorf tubes and collected by centrifugation at 2,500rpm for 5min. The cells were fixed with 2.5% glutaraldehyde for 5min at room temperature and fixed with 1% osmium tetroxide for 3h. Next, the samples were dehydrated with different concentrations of ethanol and embedded in epoxy resin, after which ultrathin sections were made and stained with 3% uranyl acetate and lead citrate. Finally, the autophagosome double-membrane structure was examined by an electron microscope.

### Immunofluorescence Staining

PK-15 cells were seeded on coverslips in six-well cell culture plates and transfected with the corresponding plasmids for 36h. Then, the cells were fixed with 4°C precooled anhydrous methanol at room temperature for 10min after washing the coverslips three times with PBS. The cells were blocked with 5% bovine serum albumin for 45min, and then a rabbit polyclonal anti-LC3B antibody, a rabbit polyclonal anti-Myc-tag antibody, or a mouse monoclonal anti-Flag-Tag antibody was added to the cells and incubated at 4°C overnight. Next, after being washed three times with PBS, the samples were incubated with the secondary antibody CoraLite488-conjugated AffiniPure goat anti-rabbit IgG (H+L; Proteintech) or Cy3-labeled goat anti-mouse IgG (H+L; Beyotime, China) at 37°C in the dark for 2h. After washing three times with cold PBS, the cell nuclei were stained with 2.5μg/ml DAPI (Sigma, D9542) in PBS for 15min at 37°C. The samples were observed using a confocal scanning laser microscope (Zeiss LSM880). PK-15 cells were transiently transfected with mRFP-eGFP-LC3 constructs to monitor autophagic flux. After 36h, the cells were treated with the indicated compounds (The autolysosome inhibitor bafilomycin A1, BAF) for 12h. All samples were analyzed using a laser scanning confocal microscope.

### Coimmunoprecipitation Assay

After PK-15 cells were cotransfected with NS5A-Flag and Myc-LC3 recombinant vectors for 48h, the cells were harvested and lysed with a mixture of RIPA buffer (Beyotime, China) and PMSF (Sangon Biotech, China). The lysates were used for coimmunoprecipitation with anti-c-Myc or anti-Flag agarose affinity gel (Thermo Fisher Scientific, 23620) following the manufacturer’s instructions. Briefly, 200μl of cell lysates were mixed with 10μl agarose slurry and incubated overnight at 4°C using 50μl positive control diluted in 150μl TBS (25mM Tris HCl, 0.15M NaCl, pH 7.2) as a positive control. The immunoprecipitates were washed with TBST three times and resuspended in 2× nonreducing sample buffer. After boiling for 5min, 2μl of 2-ME was added to the samples for Western blotting analysis with the specific antibodies.

### RNA Interference

Small interfering RNAs (siRNAs) targeting LC3 and scrambled RNA (SiLC3-NC) were purchased from GenePharma (Shanghai, China). The sequences used in this study are shown in Table 1. When PK-15 cells grown to 60–80% confluence were transfected with LC3 siRNA using TransIntro EL Transfection Reagent (TransGen Biotech, China) in six-well cell culture plates. Briefly, 4μg of siRNA and 8μl TransIntro EL were diluted in 200μl of serum-free OptiMEM medium and incubated for 15min at room temperature. The mixture was pipetted into the medium and cultured at 37°C with 5% CO_2_ for 24h. After CSFV infection for 1h, the cells were incubated for an additional 48h after the culture medium was changed.

**Table 1:** Sequence of PCR primers.

Gene name	Primer sequence (5'-3')	Note
NS5A-GFP-F	CCGAATTCTTCAAGTAATTACATACTAGAGC	Amplification of NS5A gene with GFP label
NS5A-GFP-R	ACGGATCCCAGTTTCATAGAATACACTTTTGC	
LC3-RED-F	CGGGAATTCTGATGCCCTCAGACCG	Amplification of LC3 gene with RED label
LC3-RED-R	CGGGGATCCAAGAAGCCGAAGGTTTC	
NS5A-Flag-F	ATTGGATCCATGTCAAGTAATTACATACTAGAGC	Amplification of NS5A gene with Flag tag
NS5A-Flag-R	ATTCTCGAGTCACTTATCGTCGTCATCCTTGTAATCCAGTTTCATAGAATACAC	
NS5A-804-Flag-F	ATTGGATCCATGTCAAGTAATTACATACTAGAGC	Amplification of NS5A-804 part with Flag tag
NS5A-804-Flag-R	ATTCTCGAGTCACTTATCGTCGTCATCCTTGTAATCAGCAGGCTGCAAGGTTATTTC	
NS5A-720-Flag-F	CGCGGATCCATGCCTGCCCCTTTCAGCTGTG	Amplification of NS5A-720 part with Flag tag
NS5A-720-Flag-R	ATTCTCGAGTCACTTATCGTCGTCATCCTTGTAATCAGCAGGCTGCAAGGTTATTTC	
NS5A-687-Flag-F	CGCGGATCCATGGTAGTGGTGGATACAACTGAC	Amplification of NS5A-687 part with Flag tag
NS5A-687-Flag-R	ATTCTCGAGTCACTTATCGTCGTCATCCTTGTAATCCAGTTTCATAGAATACAC	
LC3-Myc-F	ATTGGATCCATGCCCTCAGACCGGCCTTTCAAG	Amplification of LC3 gene with Myc tag
LC3-Myc-R	ATTCTCGAGTCACAGATCCTCTTCAGAGATGAGTTTCTGCTCGAAGCCGAAGGTTTCCTGGGAG	Amplification of LC3 gene with GST label
LC3-GST-F	ATTGGATCCATGCCCTCAGACCGGCCTTTCAAG	Negative control siRNA
LC3-GST-R	ATTCTCGAGTCAGAAGCCGAAGGTTTCCTGGGA	siRNA target of LC3
Si-LC3-NC-F	UUCUCCGAACGUGUCACGUTT	siRNA target of LC3
Si-LC3-NC-R	ACGUGACACGUUCGGAGAATT	siRNA target of LC3
SiLC3-1-F	CCCAGGAAACCUUCGGCUUTT	Point mutant of 15S to A
SiLC3-1-R	AAGCCGAAGGUUUCCUGGGTT	
SiLC3-2-F	UCAAGCAGCGGCGGAGCUUTT	
SiLC3-2-R	AAGCUCCGCCGCUGCUUGATT	
SiLC3-3-F	CCAUGUCAACAUGAGCGAGTT	
SiLC3-3-R	CUCGCUCAUGUUGACAUGGTT	
NS5A-15S-F	CCTGTATAAGTTCCGTGACGCTATCAAGTCTAGC	
NS5A-15S-R	GCGTCACGGAACTTATACAGGAGCTCTAGTAT	
NS5A-81S-F	CTCTTGGAGGAGGAAGGCGCATTTCTCTGCAGA	Point mutant of 81S to A
NS5A-81S-R	CGCCTTCCTCCTCCAAGAGTCTCAGCTCTCC	Point mutant of 92S to A
NS5A-92S-F	AATAAATTCGGGAGAGGTGCACGGAACTACAG	Point mutant of 274T to A
NS5A-92S-R	CACCTCTCCCGAATTTATTTCTGCAGAGAAA	Point mutant of 401T to A
NS5A-274T-F	TGCTGTAGTGGTGGATACAGCTGACGTGACCGTGA	
NS5A-274T-R	CTGTATCCACCACTACAGCAGGCTGCAAGGTTA	
NS5A-401T-F	AATTTGTTGACTACAAAGGCGCCTTTCTAACTAGA	
NS5A-401T-R	CGCCTTTGTAGTCAACAAATTTCAATAGAGCAT	

*The underline sequences represent the restriction enzyme cutting site or the Flag or Myc tag*.

### Overexpression of LC3 Mediated by Recombinant Plasmids

The LC3-Myc recombinant vector encoding LC3 was used to investigate the effect of LC3 overexpression on CSFV replication. Transfection of LC3-Myc into PK-15 cells was performed using the TransIntro EL Transfection Reagent. After 24h, the cells were infected with CSFV. At 48h postinfection, the viral genome copies were determined.

### Statistical Analysis

All experiments were conducted with at least three independent replicates, and all data are described as the mean±SD. Statistical comparisons were made using Student’s *t*-test by GraphPad Prism software. Values of *p* less than 0.05 were regarded as significant.

## Results

### CSFV NS5A Induces Autophagosome Formation

**Figure 1:**
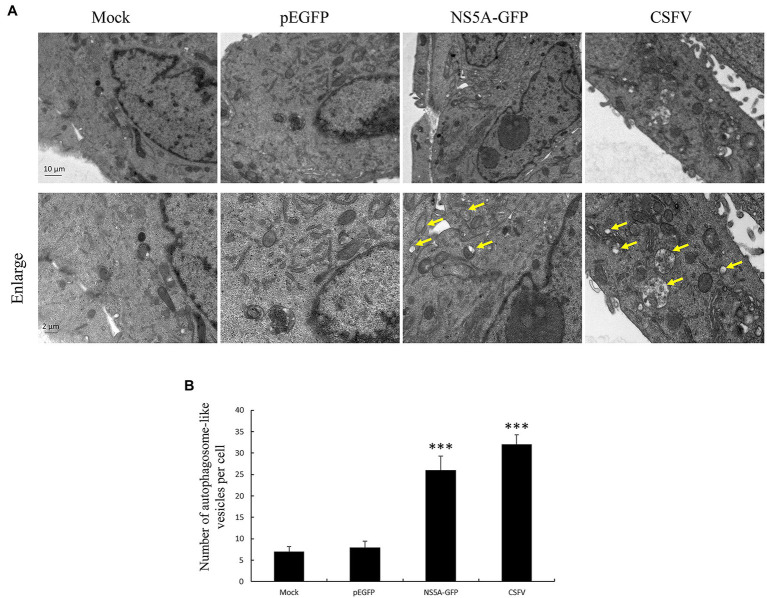
Nonstructural protein 5A (NS5A) protein raises the number of autophagosome-like vesicles. **(A)** PK-15 cells were transfected with a pEGFP or NS5A-GFP vector and mock-infected or infected with classic swine fever virus (CSFV) at an MOI of 1 for 36h, followed by electron microscopy examination. Yellow arrows indicate the structures with the characteristics of autophagosomes. **(B)** Quantification of autophagosome-like vesicles per cell image. The data represent the mean±SD of three independent experiments. Statistical comparisons were evaluated using ANOVA followed by Tukey’s HSD *post hoc* analysis. ^***^p<0.001, compared with the mock group.

It has been reported in a previous study that CSFV can induce cell autophagy (Pei et al., 2014). The mechanisms underlying this phenomenon remain unclear. NS5A is a multifunctional protein that plays a vital role in the CSFV replication cycles, participating in viral RNA synthesis and viral assembly. To determine whether the CSFV NS5A protein plays a key role in inducing cellular autophagy, we observed the number of autophagosome-like vesicles in cells expressing NS5A compared with CSFV-infected cells using transmission electron microscopy (TEM). TEM is the best direct and classical approach for observing autophagy (Ding and Yin, 2012). The results show that the number of double-membrane autophagosomes augmented in the cytoplasm of NS5A-expressing PK-15 cells, and the results were similar to those of CSFV-infected cells compared with mock- or GFP-treated cells (Figure 1A). The double-membrane vesicles were significantly increased in the NS5A-expressing cells by quantitative analysis compared with the mock or pEGFP cells (Figure 1B).

**Figure 2:**
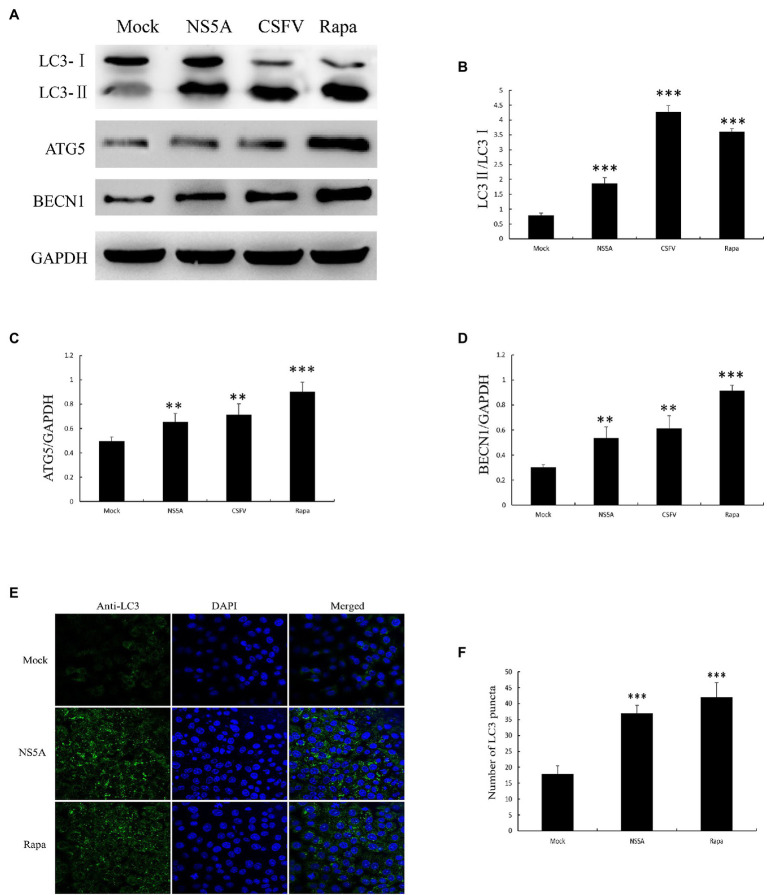
Nonstructural protein 5A expression induces cellular autophagy. **(A)** PK-15 cells were transfected for NS5A expression, infected with CSFV (MOI=1) for 36h or treated with rapamycin (Rapa, 100nM) for 48h. **(B-D)** The expression of light chain 3 (LC3), ATG5, BECN1, and GAPDH (loading control) was analyzed by immunoblotting with specific antibodies described in the Materials and Methods. **(E)** PK-15 cells were transfected with NS5A or treated with Rapa (100nM) for 48h. The cells were then fixed and processed for indirect immunofluorescence using antibodies against LC3B, followed by the corresponding secondary antibodies conjugated to FITC as described in the Materials and Methods. The cell nuclei were counterstained with DAPI. The fluorescence signals were visualized by confocal immunofluorescence microscopy. In the images, nuclear staining is shown in blue, and LC3B staining is shown in green. **(F)** The average number of LC3B puncta in each cell was determined from at least 100 cells in each group. The data represent the mean±SD of three independent experiments. Statistical comparisons were evaluated using ANOVA followed by Tukey’s HSD *post hoc* analysis. ^***^p<0.001, ^**^p<0.01, and compared with the mock group.

To further confirm that CSFV NS5A protein can trigger cellular autophagy, we detected the protein levels of autophagy markers, including LC3 conversion, ATG5, and BECN1 expression by immunoblotting. As we all known, the formation of the LC3-phosphatidylethanolamine conjugate (LC3-II) is largely used as a marker for evaluating the formation of autophagosomes (Kuma et al., 2007). In the current study, we showed that the quantity of LC3-II was significantly upregulated in NS5A-expressing cells compared to mock-infected cells, consistent with the CSFV or rapamycin treatment group (Figures 2A,B). Rapamycin (Rapa) can induce autophagy by inhibiting the mammalian target of rapamycin (mTOR) protein kinase, which was used as a positive control (Yoo et al., 2019). We next examined LC3 localization by performing indirect immunofluorescence using a rabbit polyclonal anti-LC3B antibody. We observed that the puncta of LC3 were remarkably increased compared with mock transfection (Figure 2E). We further assessed the expression of ATG5, which combines with the membranes of precursor autophagosomes (Wang and Klionsky, 2003), and BECN1, which correlate with the early steps of the autophagy pathway (Pattingre et al., 2005), by immunoblotting. NS5A-expressing cells showed increased levels of ATG5 and BECN1 compare with the mock-transfection group (Figure 2). Besides, the expression quantity of autophagy marker proteins also increased in NS5A-expressing cells in contrast to mock cells (Figures 2B–D). These results illustrate that the NS5A protein can trigger the early stages of autophagy in host cells.

### CSFV NS5A Induces Complete Autophagy

**Figure 3:**
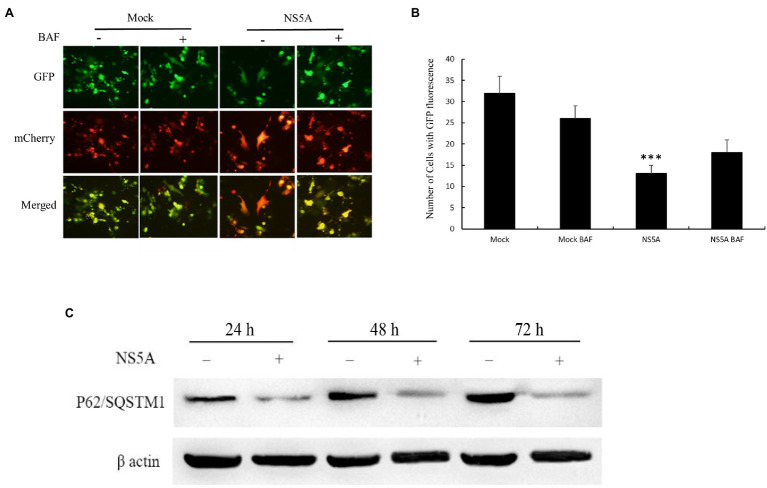
Classic swine fever virus NS5A induces complete autophagy. **(A)** PK-15 cells were seeded in six-well plates and transfected with mRFP-eGFP-LC3 only or cotransfected with NS5A and mRFP-eGFP-LC3. The cells were incubated for 48h, after which they were treated with or without 0.2μM autolysosome inhibitor bafilomycin A1 (BAF) for 12h before fluorescence imaging. **(B)** Analysis of the number of cells expressing GFP protein. **(C)** Analysis of P62/sequestosome 1 (SQSTM1) degradation in PK-15 cells transfected with or without NS5A. The cell lysates were harvested at the indicated time points and subsequently analyzed by Western blotting by probing with anti-P62/SQSTM1 antibodies. GAPDH served as a loading control for all analyses. The data represent the mean±SD of three independent experiments. Statistical comparisons were evaluated using ANOVA followed by Tukey’s HSD *post hoc* analysis. ^***^p<0.001, compared with the mock group.

The induction of autophagy by the CSFV NS5A protein has been confirmed. However, whether the CSFV NS5A protein induces autophagic flux (the fusion between autophagosomes and lysosomes) is still unclear (Jassey et al., 2019). To determine whether CSFV NS5A induces complete or incomplete autophagy, we used dual-fluorescence mRFP-eGFP-LC3, which was transfected alone or co-transfected with NS5A into PK-15 cells. RFP is used to label and track LC3. The weakening of GFP can indicate the induction of autophagolysosomes, as GFP is stable to alkaline conditions and is quenched when autophagosomes and lysosomes are fused, resulting in only red fluorescence that can be tested. The presence of autophagosomes that are not fused with lysosomes is indicated when the two fluorescent proteins are colocalized (formation of yellow puncta), whereas a red fluorescent signal indicates the fusion of autophagosomes and lysosomes, namely the completion of autophagy. After transfection for 36h, the cells were treated with the autolysosome inhibitor bafilomycin A1 (BAF) for 12h, which is a specific inhibitor of vacuolar H^+^ ATPase that can result in autophagosome accumulation by preventing fusion between autophagosomes and lysosomes (increasing yellow puncta; Yamamoto et al., 1998). As Figure 3A shows, the red puncta indicated the fusion of autophagosomes and lysosomes in mRFP-eGFP-LC3 and NS5A cotransfected cells; in other words, the expression of NS5A induced complete autophagy. The fusion between autophagosomes and lysosomes could be blocked (yellow puncta) when the autolysosome inhibitor BAF was present.

In contrast, the cells transfected with mRFP-eGFP-LC3 alone did not induce red punctate formation regardless of whether the cells were treated with BAF. As shown in Figure 3B, the number of cells with GFP fluorescence directly illustrates CSFV NS5A protein-induced complete autophagy. To further confirm these conclusions, we transfected PK-15 cells with or without NS5A and harvested the protein at 24, 48, and 72h. Western blotting analysis of P62/SQSTM1 was conducted after the protein was harvested. The P62/SQSTM1 protein is an indicator of autophagic flux and is degraded by autophagy (Bjørkøy et al., 2005). As shown in Figure 3C, the degradation of P62/SQSTM1 increased with prolonged NS5A transfection, indicating that autophagy was complete. All of these results suggest that the CSFV NS5A protein induces complete autophagy.

### CSFV NS5A Protein Interacts With Autophagy Marker of LC3

**Figure 4:**
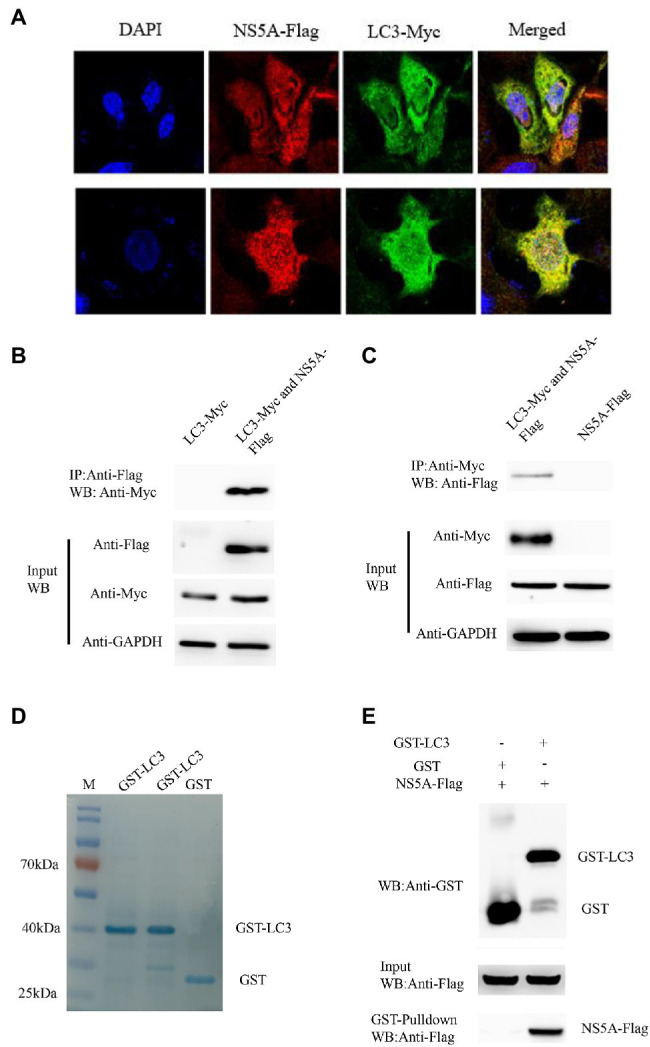
Classic swine fever virus NS5A interacts with LC3. **(A)** NS5A co-localization with LC3 protein. PK-15 cells were cotransfected with NS5A-Flag and LC3-Myc and analyzed by laser confocal microscopy after 48h. All cells were stained with DAPI. **(B)** Coimmunoprecipitation assay demonstrates that NS5A-Flag binds LC3-Myc in cotransfected cells. PK-15 cells were transfected with NS5A-Flag and LC3-Myc plasmids for 48h and harvested. Cell lysates were immunoprecipitated with an antibody against Flag, followed by Western blotting analysis. **(C)** Reciprocal co-IP experiments showed that the anti-Myc antibody precipitated NS5A-Flag. **(D)** GST and GST-LC3 are staine with Coomassie brilliant blue. **(E)** GST pulldown assay. Glutathione beads conjugated to GST or the GST-LC3 protein fusion protein were incubated with recombinant NS5A-Flag protein. After washing, proteins were eluted from the beads, and SDS-polyacrylamide gel electrophoresis (SDS-PAGE) was performed. The expression of NS5A was detected by immunoblotting with an anti-Flag mAb. GST and GST-LC3 protein expression was confirmed by immunoblotting with a rabbit anti-GST pAb.

Confocal microscopy was performed to observe the subcellular localization of NS5A and LC3 in PK-15 cells to determine the co-localization of NS5A and LC3. We expressed full-length NS5A tagged with Flag and LC3 tagged with Myc in PK-15 cells. Indirect immunofluorescence was used to detect the co-localization between NS5A and LC3. After incubation with the secondary antibodies [Cora Lite 488-conjugated Affini Pure goat anti-rabbit IgG (H+L) and Cy3-labeled goat anti-mouse IgG (H+L) for detection of the Flag and Myc tags, respectively], the distribution of NS5A and LC3 was observed by confocal microscopy. As Figure 4A shows, apparent co-localization of NS5A with cellular LC3 was observed in NS5A-overexpressing cells (yellow puncta).

To further verify the interaction between NS5A and LC3, coimmunoprecipitation experiments were performed to verify whether the NS5A protein interacts with LC3. The NS5A-Flag and Myc-LC3 plasmids were cotransfected into PK-15 for 36h, and the cell lysates were co-immunoprecipitated with anti-Flag affinity gel. Finally, the proteins in the complexes were detected by Western blotting. The results showed that NS5A-Flag interacted with the LC3-Myc protein (Figure 4B). In the reciprocal co-IP assay, the results also showed that the anti-Myc antibody precipitated NS5A-Flag (Figure 4C). These results suggest that an *in vivo* interaction between NS5A and LC3 exists in cells.

The GST pull-down assay was performed to confirm the interaction between NS5A and LC3 further. Recombinant full-length LC3-GST fusion protein and GST protein were inducibly expressed in bacteria of *Escherichia coli* BL21 (DE3) Strain (Figure 4D), and NS5A-Flag was transfected into PK-15 cells, respectively. The relationship between NS5A and LC3 was tested by GST pulldown assay. As shown in Figure 4E, NS5A-Flag was detected in an LC3-GST complex. These results illustrate that LC3 can interact with NS5A *in vitro*.

### The N-Terminus of the NS5A Protein Is a Key Functional Area for Inducing Autophagy

**Figure 5:**
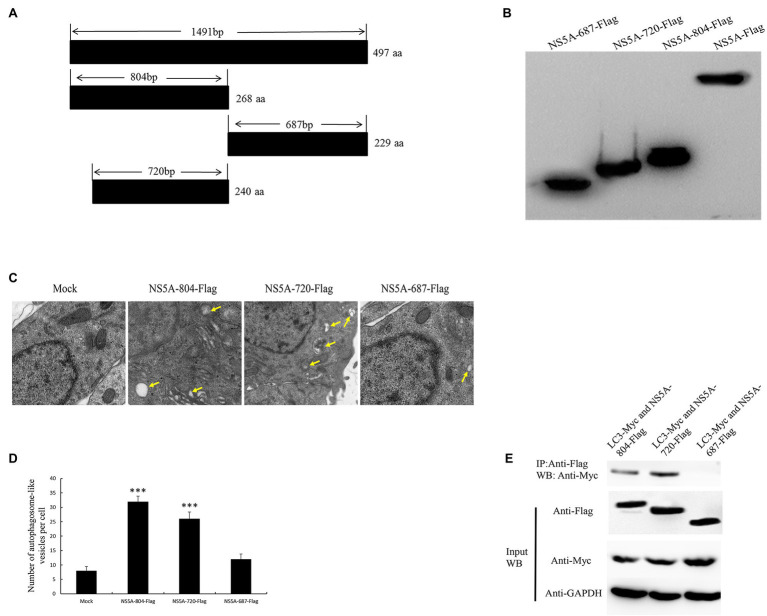
The N-terminal region of NS5A increases the formation of autophagosome-like vesicles and is required for association with the LC3 protein. **(A)** Schematic representation of the protein domains of the CSFV NS5A protein. **(B)** Western blotting with anti-Flag antibody. **(C)** PK-15 cells were transfected with different parts of the NS5A-Flag vector and studied by electron microscopy. Yellow arrows indicate the structures with the characteristics of autophagosomes. **(D)** Quantification of autophagosome-like vesicles per cell image. The average number of vesicles in each cell was obtained from at least 10 cells undergoing each treatment. **(E)** The N-terminal region of CSFV NS5A interacts with LC3. PK-15 cells were transfected with different parts of the NS5A-Flag and LC3-Myc plasmids for 48h and harvested. Cell lysates were immunoprecipitated with an antibody against Flag followed by Western blotting analysis. The data represent the mean±SD of three independent experiments. Statistical comparisons were evaluated using ANOVA followed by Tukey’s HSD *post hoc* analysis. ^***^p<0.001 compared with the mock group.

**Figure 6:**
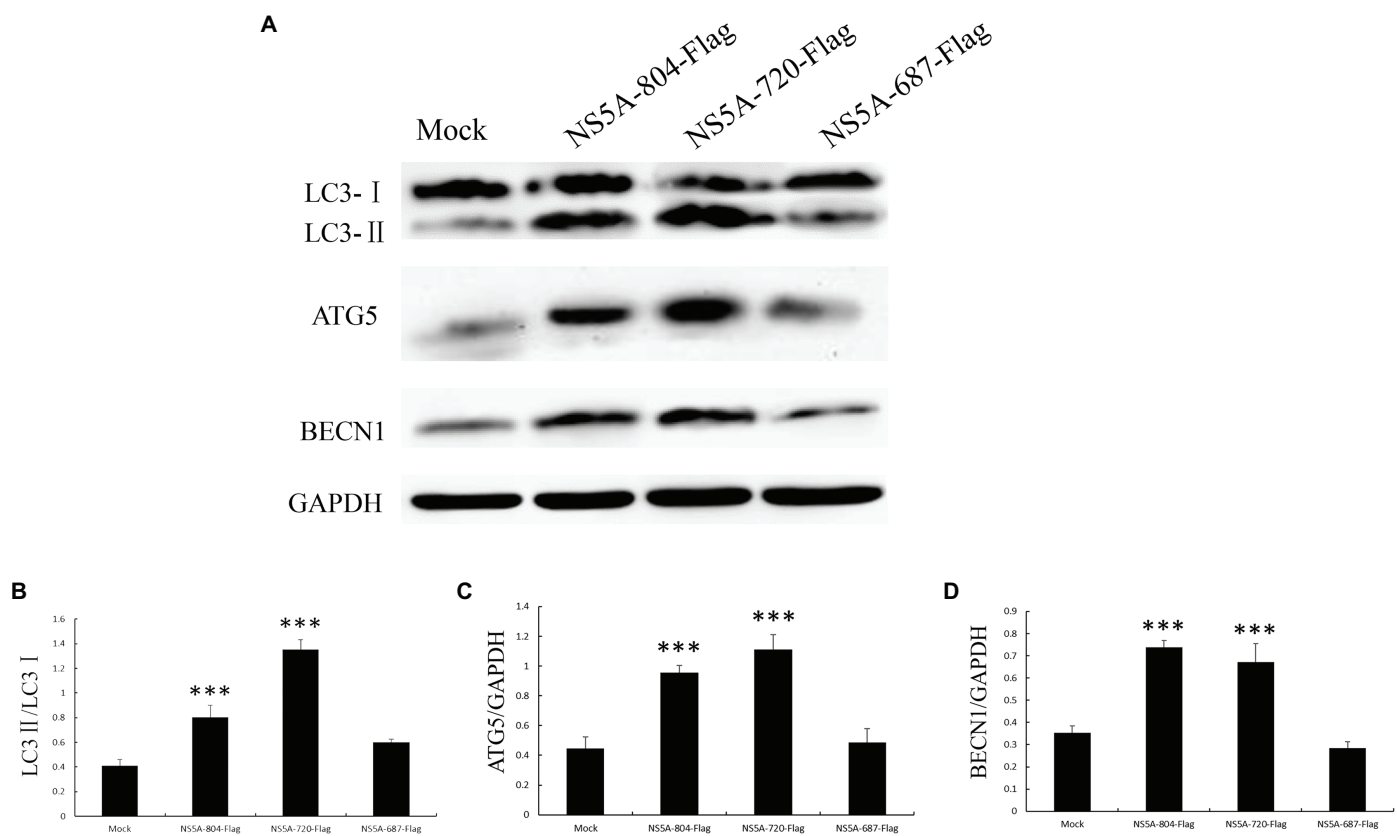
Expression of autophagy marker proteins in cells expressing different portions of the NS5A gene. **(A)** PK-15 cells were transfected with sequences expressing different portions of the NS5A gene or a mock plasmid. The expression of LC3, ATG5, BECN1, and GAPDH (loading control) was analyzed by immunoblotting with specific antibodies described in the Materials and Methods. The data represent the mean±SD of three independent experiments. Statistical comparisons were evaluated using ANOVA followed by Tukey’s HSD *post hoc* analysis. ^***^p<0.001, compared with the mock group.

The amino acid function of the NS5A protein was analyzed through the online analyses of protein informatics websites (http://smart.embl-heidelberg.de and http://www.expasy.org/). This analysis predicted that the first 84 nucleotides of the CSFV NS5A gene are membrane localization sequences and a low repetitive sequence near nucleotide 804. Based on this, the NS5A protein was divided into three functional regions (NS5A-804, NS5A-720, and NS5A-687), and these three gene sequences were inserted into the eukaryotic plasmid pcDNA3.1(+) with a Flag tag (Figures 5A,B). After transfection into PK-15 cells for 48h, autophagosome-like vesicles were significantly increased in NS5A-804- and NS5A-720-transfected cells (Figures 5C,D). Interestingly, NS5A-804 and NS5A-720 interacted with LC3, as shown by the results of the co-IP experiment (Figure 5E). Consistently, the expression of the autophagy-related proteins LC3, BECN1, and ATG5 was detected by Western blotting. All of our results showed that the expression of LC3, ATG5, and BECN1 was increased in NS5A-804- and NS5A-720-transfected cells (Figure 6); in other words, the N-terminus of the NS5A protein is a key functional area for inducing autophagy and mediating interaction with the LC3 protein.

### The N-terminal Amino Acids Serine 81 and Serine 92 of the NS5A Protein Play an Important Role in Cell Autophagy

**Figure 7:**
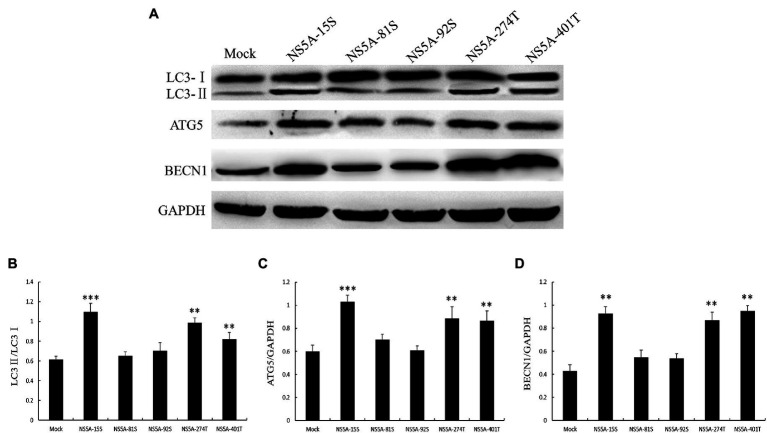
The key amino acids of NS5A affect the expression of autophagy marker proteins. **(A)** PK-15 cells were transfected with constructs expressing NS5A with specific amino acid substitutions. The expression of LC3, ATG5, BECN1, and GAPDH (loading control) was analyzed by immunoblot with specific antibodies described in the Materials and Methods. The data represent the mean±SD of three independent experiments. Statistical comparisons were evaluated using ANOVA followed by Tukey’s HSD *post hoc* analysis. ^***^p<0.001, ^**^p<0.01, compared with the mock group.

To further investigate which amino acids in the NS5A protein play a role in inducing autophagy; we selected five key phosphorylated amino acids, serine 15 (15S), serine 81 (81S), serine 92 (92S), threonine 274 (274T), and threonine 401 (401T), for a site-specific mutation to alanine. We transfected PK-15 cells with these mutated sequences and examined the expression of the autophagy-related proteins LC3, ATG5, and BECN1. As shown in Figure 7, serine 81 and serine 92 of the NS5A protein play a critical role in the process of NS5A-induced autophagy.

### LC3 Enhances CSFV Proliferation

**Figure 8:**
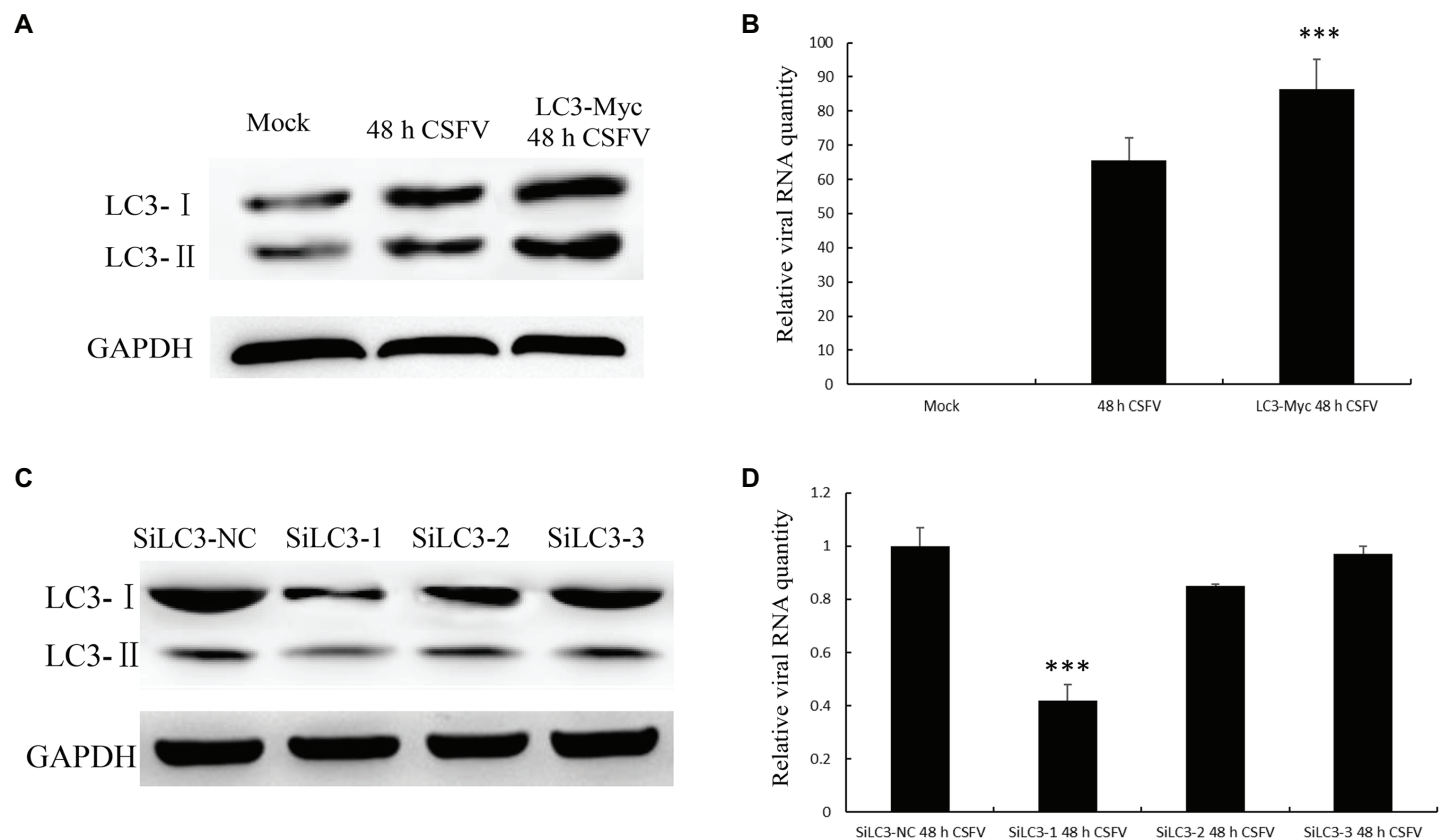
Light chain 3 promotes CSFV propagation. **(A)** LC3 overexpression and CSFV infection showed changes in the protein level of LC3. **(B)** The change in CSFV RNA expression level when LC3 was overexpressed. **(C)** Knockdown of LC3, mediated by small interfering RNA (siRNA), showed changes in LC3 expression levels. **(D)** The change in CSFV RNA expression level when LC3 expression was knocked down. The data represent the mean±SD of three independent experiments. Statistical comparisons were evaluated using ANOVA followed by Tukey’s HSD *post hoc* analysis. ^***^p<0.001 compared with the mock group.

To further study the effect of autophagy on CSFV replication, LC3 was successfully overexpressed in PK-15 cells, as detected by Western blotting (Figure 8A). Then, the transfected cells were infected with CSFV for 48h. The qPCR results showed an increased level of CSFV mRNA in LC3-overexpressing cells (Figure 8B). Conversely, we also investigated whether the suppression of LC3 expression can reduce the CSFV mRNA level in PK-15 cells transfected with LC3-shRNA. As shown in Figures 8C,D, compared with the mock group, the knockdown of LC3 protein significantly reduced the expression of viral mRNA. All of these findings indicate that autophagy has a positive role in CSFV proliferation.

## Discussion

Classic swine fever virus, like all other positive-stranded RNA viruses, utilizes intracellular membrane structures to its replication (Wang et al., 1999; Pei et al., 2014). CSFV infection has been reported to induce autophagy and promote viral proliferation *in vivo* and *in vitro* (Pei et al., 2014; Gou et al., 2017). Nevertheless, the specific viral protein and the associated mechanism by which these membrane proteins mediate this process during CSFV replication in host cells have not been elucidated. In the current study, we firstly illustrate that the CSFV NS5A protein can induce autophagy *in vitro*. We also show that NS5A can interact with the LC3 protein and that LC3 promotes CSFV replication.

Autophagy has been reported to correlate with immunity by directly regulating pattern recognition receptor-mediated type I interferon production or by conducing to antigen presentation (Levine and Deretic, 2007; Shaw et al., 2013; Bendorius et al., 2018). Autophagy is induced and participates in CSFV replication, but the mechanism is not clearly understood. We analyzed whether the NS5A protein plays a significant role in inducing autophagy *in vitro* in the PK-15 cell line, a common model cell line for studying CSFV infection (Grummer et al., 2006; Zhang et al., 2021).

When PK-15 cells were transfected with NS5A, we found that the number of autophagosome-like vesicles in target cells was similar to that seen in CSFV infection by TEM (Figure 1). This phenomenon was further verified by immunoblotting analysis showing an upregulate expression of autophagic proteins in target cells, with NS5A inducing a trend similar to CSFV infection or Rapa treatment (Figure 2). Further study showed that NS5A increased and redistributed the autophagy marker LC3 in host cells (Figure 2). These data imply that cellular autophagy can be induced by NS5A protein. Recent researches have testified that the marker of autophagy not only augment autophagosome formation and the upregulate of specific proteins but also appears the autophagic flux, which can be observed the expression levels of P62/SQSTM1 in the condition of lysosome inhibitors BAF treated or not (Pankiv et al., 2007). As shown in Figure 3, CSFV NS5A could induce the autophagic flux, and NS5A significantly promoted the degradation of P62/SQSTM1 compared with mock treatment (Figure 3C). These data illustrate that NS5A as a replicase of CSFV can enhance autophagic flux and induce a complete autophagic response in the host cells, ulteriorly explaining how the NS5A protein regulates cellular signal pathways, which in turn play a role in the process for favoring viral replication and persistence.

It has been previously reported that virus RNA replication complexes were located in the autophagosome membrane, promoting viral replication (Lee et al., 2008; Wong et al., 2008). As an important component of the autophagosome membrane, LC3 is largely used autophagy marker to evaluate the formation of autolysosomes. To further understand how autophagy is influenced by CSFV NS5A protein, we analyzed the colocalization of LC3-II with the NS5A protein. The results showed that NS5A strengthened the redistribution and increased expression of LC3 (Figure 4A), and interestingly, LC3 enhanced the viral replication level when overexpressed (Figure 8). These results indicate that the membranes of autophagosome-like vesicles were beneficial for CSFV proliferation. Our results, therefore, indicate a new function to add to the list of NS5A’s known multiple functions in adjusting cellular pathways and prominent the role of LC3 in facilitating CSFV replication.

Classic swine fever virus tends to cause chronic infection partly due to its plentiful immune escape strategies; for example, CSFV not merely uses the autophagosome as a self-replication site to promote its RNA replication, but also to suppress the host immune response and establish a long-term infection (Fan et al., 2019). Recently, CSFV-induced autophagy has been reported to take part in the innate immune evasion of the virus, which could be related to the induction of autophagic flux (Pei et al., 2014). Herein, we proved that CSFV NS5A could induce a complete autophagic response by interacting with LC3 to promote viral replication, reminding us that NS5A could promote CSFV replication by weakening innate immune signaling through autophagy to trigger the recycling of cellular proteins.

In conclusion, we illustrated that CSFV NS5A firstly trigger complete autophagy while interacting with the LC3 protein and promoting viral replication. Furthermore, the N-terminal region and the phosphorylation of amino acids 81 and 92 of the NS5A protein are essential for inducing autophagy. This study provides new insights that implicate NS5A particularly its N-terminal amino acids serine 81 and serine 92 as an important regulator to adjust the cellular autophagy during the CSFV infection. Reveal the mechanism of autophagy induced by NS5A and its interaction with LC3 may enhance our understanding of CSFV pathogenesis and identify novel targets for therapeutic the disease.

## Data Availability Statement

The original contributions presented in the study are included in the article/supplementary material, further inquiries can be directed to the corresponding author.

## Author Contributions

CZ and XZ conceptualized the experiments. XW, JS, and MG performed the experiments. XZ analyzed the results. CZ and YW wrote the paper. All authors contributed to the article and approved the submitted version.

## Conflict of Interest

The authors declare that the research was conducted in the absence of any commercial or financial relationships that could be construed as a potential conflict of interest.

## Publisher’s Note

All claims expressed in this article are solely those of the authors and do not necessarily represent those of their affiliated organizations, or those of the publisher, the editors and the reviewers. Any product that may be evaluated in this article, or claim that may be made by its manufacturer, is not guaranteed or endorsed by the publisher.
